# Retraction: Molecular characterization of *Legionella pneumophila*-induced interleukin-8 expression in T cells

**DOI:** 10.1186/1471-2180-11-127

**Published:** 2011-06-02

**Authors:** Reika Takamatsu, Hiromitsu Teruya, Eriko Takeshima, Chie Ishikawa, Kunihiro Matsumoto, Naofumi Mukaida, Jian-Dong Li, Klaus Heuner, Futoshi Higa, Jiro Fujita, Naoki Mori

**Affiliations:** 1 Division of Molecular Virology and Oncology, Graduate School of Medicine, University of the Ryukyus, 207 Uehara, Nishihara, Okinawa 903-0215, Japan; 2 Division of Control and Prevention of Infectious Diseases, Graduate School of Medicine, University of the Ryukyus, 207 Uehara, Nishihara, Okinawa 903-0215, Japan; 3 Transdisciplinary Research Organization for Subtropics and Island Studies, University of the Ryukyus, 1 Senbaru, Nishihara, Okinawa 903-0215, Japan; 4 Department of Molecular Biology, Graduate School of Science, Nagoya University, Chikusa-ku, Nagoya 464-8602, Japan; 5 Division of Molecular Bioregulation, Cancer Research Institute, Kanazawa University, 13-1 Takara-machi, Kanazawa 920-0934, Japan; 6 Department of Microbiology and Immunology, University of Rochester Medical Center, 601 Elmwood Avenue, Rochester, New York 14642, USA; 7 Project group 26 "Nosocomial Infections of the Elderly", Robert Koch-Institut, 20 Nordufer, Berlin 13353, Germany

## Abstract

Article Retracted

## Retraction

After lengthy investigation by the editors, the original article [1] has been retracted because of inappropriate duplication of images from previously published articles. The last author, Naoki Mori takes full responsibility and apologizes for any inconvenience caused.
